# Naming and conceptual understanding in frontotemporal dementia

**DOI:** 10.1016/j.cortex.2019.04.027

**Published:** 2019-11

**Authors:** Julie S. Snowden, Jennifer M. Harris, Jennifer A. Saxon, Jennifer C. Thompson, Anna M. Richardson, Matthew Jones, Christopher Kobylecki

**Affiliations:** a Cerebral Function Unit, Manchester Centre for Clinical Neurosciences, Salford Royal NHS Foundation Trust, Salford, UK; b Division of Neuroscience and Experimental Psychology, Faculty of Biology, Medicine and Health, University of Manchester, Manchester, UK

**Keywords:** Frontotemporal dementia, Naming, Temporal lobes, Imaging, Semantic hub

## Abstract

Behavioural variant frontotemporal dementia (bvFTD) is characterised by behaviour change and impaired executive skills. There is growing evidence that naming difficulties may also be present but the basis for these is unclear. A primary semantic deficit has been proposed, although executive contributions to naming breakdown are also possible. The study aimed to improve understanding of the naming disorder in bvFTD through direct comparison with semantic dementia (SD), and examination of neural correlates. It aimed also to address current controversies about the role of the anterior temporal lobes in semantic memory. We studied 71 bvFTD and 32 SD patients. Naming data were elicited by two picture naming tests (one challenging and one less demanding) and word comprehension by word-picture matching. Structural magnetic resonance images were rated blind using a standardised visual rating scale. Around half of bvFTD patients showed impaired naming and 17% impaired word-picture matching. Deficits in bvFTD were less severe than in SD, but showed a similar pattern. There were strong inverse correlations between naming scores and atrophy in temporal structures, particularly temporal pole and fusiform gyrus. Word comprehension scores correlated more strongly with posterior than anterior temporal lobe atrophy in SD. Error analysis highlighted a significant relationship in both groups between associative-type responses and temporal pole atrophy. By contrast, ‘don't know’ responses, suggesting a loss of conceptual knowledge, correlated with more posterior temporal regions. There was some correlation in bvFTD between naming and executive test performance but not with frontal lobe atrophy. The findings support the view that naming problems can arise in bvFTD independently of patients' ‘frontal’ executive impairment and highlight clinical overlap between bvFTD and SD. We discuss the findings in relation to the hub and spoke model of semantic memory and argue against the notion of an anterior temporal lobe semantic hub.

## 1 Introduction

Behavioural changes and impaired executive skills are the defining features of the behavioural form of frontotemporal dementia (bvFTD) (Neary et al., 1998, Rascovsky et al., 2011). Nevertheless, additional symptoms may be present that overlap with those of primary progressive aphasias (Blair et al., 2007, Hardy et al., 2015, Harris et al., 2016), which share a related frontotemporal lobar degeneration pathology. Problems in naming are particularly prominent (Blair et al., 2007, Grossman et al., 2004, Hardy et al., 2015, McMillan et al., 2004). They have been identified in bvFTD early in the course of disease (Ranasinghe et al., 2016), and even in the presymptomatic phase (Rohrer et al., 2015). In an international study of genetic FTD (Rohrer et al., 2015), pre-symptomatic mutation carriers showed significantly reduced naming performance compared to non-carriers up to five years prior to their predicted clinical onset of illness.

The basis for naming problems in bvFTD is open to question. It is plausible that they might arise as a secondary consequence of behavioural/executive change, for example, as a result of impulsivity, inattention, economy of effort and lack of concern for accuracy or because of difficulties in response inhibition leading to response perseveration. On the other hand, given that bvFTD involves temporal as well as frontal lobes, naming problems might have a primary linguistic basis and occur as an independent accompaniment to patients' behavioural/executive disorder. Some studies report language problems in bvFTD that are qualitatively similar to those of the progressive aphasias, albeit less severe, implying a primary linguistic deficit and have argued for a core semantic deficit in bvFTD (Hardy et al., 2015). However, the possible contribution to naming of executive factors has also been acknowledged (Grossman et al., 2004). In keeping with this, a comparative study of speech fluency in patients with progressive nonfluent aphasia and bvFTD, showed that whereas grammatical measures predicted the number of words produced per minute in speech samples of non-fluent aphasia patients only executive measures were significant predictors in bvFTD (Gunawardena et al., 2010). Studies of object and action naming indicate disproportionate impairment of action naming in bvFTD compared to Alzheimer's disease (Cappa et al., 1998, Silveri et al., 2003) and semantic dementia (SD) (Cotelli et al., 2006), which has been ascribed to the greater executive demands in naming verbs (Silveri et al., 2003).

A primary semantic deficit underpinning naming deficits in bvFTD might be expected to be associated with parallel problems in word comprehension. In the study by Hardy et al. (2015) comprehension difficulties, determined by a concrete word synonym judgement task, were indeed identified. However, others have found no deficit in bvFTD using the same task (Hsieh et al., 2012). Clinical variability, differences in group size (24 *vs* 8), and genetic factors might all contribute to differences in findings.

Interpretation of naming problems in bvFTD is complicated. There is wide clinical heterogeneity within FTD so that factors influencing naming performance may not pertain equally to all patients. Indeed, in a retrospective study of 185 bvFTD patients (Saxon et al., 2017) impairments in confrontation naming were identified in 54%, suggesting that not all patients are affected. Similarly, in the study of 24 bvFTD patients by Hardy et al. (2015) ten patients performed more than two standard deviations below controls on tests of naming and word comprehension, whereas 14 did not. Genetic mutations in FTD are known to influence clinical phenotype (Boeve et al., 2012, Snowden Rollinson et al., 2012, Van Langenhove et al., 2013) and correspondingly, to have distinct neuroimaging signatures (Rohrer et al., 2010, Whitwell et al., 2012). Semantic deficits have been identified in particular in association with mutations in the microtubule associated protein tau (*MAPT)* gene (Janssen et al., 2002, Pickering-Brown et al., 2002), occurring with greater frequency than with progranulin (*GRN)* mutations or with repeat expansions in the chromosome 9 open reading frame (*C9orf72)* gene (Hardy et al., 2015, Snowden et al., 2015).

In keeping with those findings Rohrer et al. (2015) found, in their study of the pre-clinical phase of genetic FTD, reductions in naming performance to be most marked in *MAPT* mutation carriers. Many existing studies of naming in bvFTD involve small numbers of participants, which, in view of the inherent clinical heterogeneity, might not be representative of the population of bvFTD. There is a need for a systematic investigation of naming in a large patient cohort.

A valuable means of exploring naming is to examine the inter-relationship with other cognitive measures. Grossman et al. (2004) and McMillan et al. (2004) distinguished between a lexical retrieval and semantic contribution to naming in bvFTD, based on the association between confrontation naming scores and measures of category fluency and semantic category judgement. Consistent with those findings they identified significant correlations between grey matter volume in both bilateral anterior temporal and frontal regions. Hardy et al. (2015) identified neuroanatomical correlates of naming and word comprehension in bvFTD in inferior frontal and anterior-inferior temporal cortices within the dominant hemisphere language network.

A complementary but under-explored approach to understanding naming in bvFTD is to examine the nature of naming errors. In a previous study of naming in SD (Snowden et al., 2018) we identified distinct error patterns as a function of left versus right and anterior versus posterior temporal lobe atrophy, providing evidence of their explanatory value. In view of claims that naming in bvFTD incorporates a significant semantic component it would be important to know the extent to which error profiles in bvFTD mirror those of patients with SD or are qualitatively distinct.

A primary purpose of the current study was to better characterise the naming disorder in bvFTD. Examination of the relationship between naming and word comprehension performance and a) executive measures and b) ratings of atrophy in different regions of the frontal and temporal lobes ought to shed light on the basis of these difficulties in bvFTD. A direct comparison of performance with that of patients with SD and an analysis of naming errors would enable us to determine whether patients’ naming disorder is similar to or different from the prototypical semantic impairment of SD. It would determine too whether there is phenotypic variation within bvFTD with respect to the nature of naming disorder. If there is variation within bvFTD then the study offers the potential to explore possible contributory factors, such as family history, genetic mutation and distribution of atrophy.

Aside from its clinically motivated aim the study sought to address a more theoretical issue: the role of the anterior temporal lobes in semantic memory. The profound, multimodal loss of conceptual knowledge seen in SD, occurring in the context of severe atrophy of the anterior temporal lobes, led to the influential view of the anterior temporal lobes as a ‘semantic hub’ (Patterson, Nestor, & Rogers, 2007), an area of convergence in which concepts are represented in amodal form. The notion of amodal representation has been challenged by studies that have shown differential breakdown in knowledge for words, objects and faces as a function of the relative preponderance of atrophy in the left and right hemispheres (Gainotti, 2007, Gainotti et al., 2003, Gainotti et al., 2010, Snowden et al., 2004, Snowden Thompson et al., 2012). To reconcile such findings the conceptualisation of the putative hub has undergone progressive revision (Hoffman et al., 2012, Lambon Ralph et al., 2017, Rice et al., 2015) leading to it being framed as a ‘transmodal’ hub in which there is pan-category semantic representation, supported jointly by left and right anterior temporal lobes, but also subtle functional gradations between and within the anterior temporal lobes, which emerge as a consequence of differential connectivity with primary sensory, motor and limbic regions. There is, moreover, increased emphasis on the ‘spokes’ as opposed to the ‘hub’ component of the model of semantic memory (Gainotti, 2017, Woollams and Patterson, 2018). Nevertheless, the precise role of the anterior temporal lobes and the status of a representational hub remain areas of contention. In our previous study of SD patients (Snowden et al., 2018) whereas naming correlated strongly with anterior temporal lobe atrophy, comprehension of words correlated more strongly with posterior temporal lobe atrophy. Such findings would argue against the notion of the anterior temporal lobes as semantic hub. A study of naming and word comprehension in bvFTD has the potential to inform the debate.

## 2 Methods

This is a retrospective study of clinical data acquired during patients’ routine diagnostic or follow-up assessments. We report how we determined our sample size, all data exclusions, all inclusion/exclusion criteria, whether inclusion/exclusion criteria were established prior to data analysis, all manipulations and all measures in the study.

### 2.1 Participants

All participants were patients who had been assessed between 2007 and 2017 in a diagnostic neuroscience unit specialising in early onset and atypical dementias.

The key group of interest were patients clinically diagnosed with behavioural variant of frontotemporal dementia (bvFTD). Patients were included in the study only if i) they fulfilled contemporary consensus criteria for bvFTD on the basis of their behavioural disorder and accompanying executive deficits (Rascovsky et al., 2011) i.e., they had a progressive history of behavioural and cognitive change, consisting of at least three of the following: early behavioural disinhibition, early apathy or inertia, early loss of sympathy or empathy, early perseverative, stereotyped or compulsive/ritualistic behaviour, hyperorality and dietary change, executive deficits with relative sparing of memory and visuospatial deficits ii) they had undergone comprehensive neuropsychological assessment as part of their diagnostic and follow-up clinical evaluation and data were available from picture naming and word-picture matching tests and iii) structural magnetic resonance imaging scans were available and amenable to rating using a well-established visual rating scale.

Patients were excluded if i) the clinical history raised the possibility of an alternative or mixed aetiology to account for their behavioural symptoms (e.g., history of alcohol abuse, vascular disease, head injury etc), or their symptoms could be accounted for by a psychiatric diagnosis or other medical disorder ii) follow-up testing showed no evidence of progression raising the possibility of ‘FTD phenocopy’ syndrome, iii) patients' clinical condition was sufficiently advanced at the time of the scan to render them formally untestable, and iv) there was a temporal discrepancy between clinical assessment and brain scan dates exceeding 12 months. In the majority of cases neuroimaging was carried out within weeks of the clinical evaluation.

Patients with SD were included in the study as a reference group. All patients exhibited a multimodal disorder of semantic knowledge and fulfilled criteria for SD (Neary et al., 1998). Most also fulfilled criteria for semantic variant primary progressive aphasia (Gorno-Tempini et al., 2011) although in some patients with more right than left-sided atrophy the earliest presenting symptom was difficulty in face recognition rather than in the language domain. All patients had a clinical history and evidence on cognitive examination of problems in both naming and word comprehension. Like the bvFTD group, patients were included in the study only if i) they had undergone clinical neuropsychological evaluation that included picture naming and word picture-matching tests and ii) neuroimaging had been undertaken close to the time of patients’ clinical assessment, which supported the clinical diagnosis.

For most participants in both diagnostic groups brain scanning had been undertaken for clinical purposes as part of the patient's diagnostic work up, although in a minority of cases research scans were available. Patients, or their consultees, had provided written consent for clinical data to be used for research purposes. Ethical approval had been obtained for the clinical research database (NREC reference: 09/h0906/53 + 5). Where research scans had been undertaken independent consent procedures applied.

### 2.2 Background clinical data

Demographic and clinical data extracted from patients' clinical records included participants’ gender, handedness, age at onset of symptoms, duration of symptoms at test, educational background, the presence of a family history of dementia, the outcome, where applicable, of screening for genetic mutations and neurological signs.

Background cognitive data included Mini Mental State Examination (MMSE) scores (Folstein, Folstein, & McHugh, 1975) at the time of testing, and scores on letter fluency (the number of words beginning with F, A and S each in one minute), category fluency (the number of animals produced in one minute), Weigls’ block sorting test (requiring grouping of 12 coloured blocks into according to three sorting rules: colour, shape and motif, maximum score 9) and Brixton Spatial Anticipation test, a nonverbal test of rule detection and mental set shifting (Burgess & Shallice, 1996).

### 2.3 Naming and comprehension tasks

Naming was assessed by means of two picture naming tests: the Graded Naming test (McKenna & Warrington, 1980) and an easier locally constructed test (Manchester naming test). The Graded Naming test comprises 30 items of increasing difficulty and is sufficiently demanding that it typically elicits floor level performance in patients with SD. The locally constructed test is a 40-item test that uses pictures drawn from the corpus of Snodgrass and Vanderwart (1980) and consists of 10 items from each of the following semantic categories: animals, fruits/vegetables, articles of clothing, household objects. The category sets are matched for word frequency and age of acquisition, but clothing and objects are rated as more familiar than the animals and vegetables. The naming test, which is appropriately pitched for patients with SD, is sufficiently easy to yield ceiling or close to ceiling level performance in healthy controls. Word comprehension was assessed by a word-picture matching test involving the same 40 items as the naming test, permitting direct comparison of naming and comprehension scores. The participant is required to match a printed word with one of four semantically related pictures. The location of the target picture (top-left, top-right, bottom-left, bottom-right) is balanced across the 40 items. Naming tests were administered first. The word-picture matching test was administered separately: all items were administered regardless of whether items had been correctly named. Performance on naming and comprehension tasks was measured in terms of the number of correct responses, in total and for biological and non-biological semantic categories. In addition, incorrect naming responses were classified with respect to the nature of errors.

#### 2.3.1 Classification of naming errors

A classification system was applied in line with that used in a previous study of naming in SD (Snowden et al., 2018).

**Table:** 

Semantic errors (coordinate category)	e.g., ‘dog’ for rabbit
	e.g., ‘banana’ for apple
Superordinate category substitutions	e.g., ‘animal’ for rabbit
Associative/functional/gestural	e.g., ‘when it rains’ for umbrella
	e.g., ‘in Australia’ for kangaroo
	e.g., hammering action for hammer
Visually related misidentifications	e.g., ‘hat’ for mushroom
Omissions/vague or irrelevant responses	e.g., ‘don't know’ or ‘I like that’
Acceptable alternative responses	e.g., ‘coat’ for jacket

Responses were classed as ‘acceptable’ alternatives if they are produced by healthy controls, as specified by normative data (e.g., Snodgrass & Vanderwart, 1980), or are locally acceptable synonyms (e.g., ‘brolly’ in place of umbrella). The boundaries between what was considered acceptable and non-acceptable were determined by consensus and checks were subsequently made to ensure consistency of application of classification labels across all participants. Error classification was carried out without knowledge of the image analysis.

Naming responses were initially recorded verbatim on a database to facilitate the coding of naming errors and to ensure consistency of classification across the cohort.

### 2.4 Imaging

Patients’ magnetic resonance scans (coronal TI or FLAIR images) were rated for severity of atrophy using a visual rating scale that has been specifically designed to measure atrophy in frontal and temporal lobe structures (Davies et al., 2009) and so is particularly applicable for evaluation of FTD. The Davies method uses a five-point scale, ranging from no atrophy (0) to severe atrophy (4). It distinguishes between sub-regions of the anterior brain in each hemisphere: temporal pole, anterior basal ganglia, orbitofrontal gyrus, lateral frontal gyrus, anterior cingulate gyrus, anterior hippocampus, anterior parahippocampal gyrus (entorhinal), collateral sulcus (perirhinal), anterior fusiform gyrus, lateral temporal gyrus, insula, mid hippocampus, superior temporal gyrus, posterior hippocampus and posterior temporal gyrus. This visual rating scale was considered optimal. Voxel-based morphometric analysis was not viable, as scans had been carried out in different clinical centres so data acquisition protocols were not identical for all patients. The visual rating scale has been demonstrated to show good inter-rater reliability, strong correlations between atrophy scores and voxel based morphometric analyses and excellent differentiation between frontotemporal lobar degeneration and other forms of degenerative dementia (Davies et al., 2009, Harper et al., 2016). Harper et al. (2016) have argued for the utility of visual rating scales in improving diagnostic accuracy.

To enable direct comparison with findings from an earlier study (Snowden et al., 2018), MR images were rated also using a simpler rating scale (Kipps et al., 2007), which yields separate ratings for frontal, anterior temporal and posterior temporal lobes in left and right hemispheres. The relationship between the Kipps and Davies scales is presented as Supplementary material.

Ratings were carried out in blinded fashion by CK, a specialist neurologist who had training and substantial experience of image analysis. CK had no knowledge of the patients, no access to their cognitive data, and no knowledge of their clinical scan reports, to eliminate potential biases in rating.

### 2.5 Statistical analysis

Group comparisons were made using *t*-tests, repeat measures analysis of variance, Mann–Whitney tests or, chi-squared tests, depending on the interval, ordinal or categorical nature of the data. Correlative analyses used Pearson's R for parametric or Spearmans correlations for non-parametric data. Correlations between language performance and atrophy ratings involved multiple comparisons for each analysis (30 for the Davies scale). False discovery rates for those analyses were controlled using the Benjamini-Hochberg procedure (Benjamini and Hochberg, 1995), using a false discovery rate (FDR) of .05. In this procedure raw *p* values are ranked (smallest first) and compared to their Benjamini-Hochberg critical value, calculated using the formula (i/m)Q where i is the rank, m is the total number of tests (30) and W is the false discovery rate (*p* = .05). The largest *p* value that has *p* < (i/m)Q determines the cut-off for significance. For most analyses *p* values < .008 were identified as significant. Values between *p* = .008 and *p* = .01 were regarded as trends.

## 3 Results

### 3.1 Clinical characteristics

Seventy-one patients with bvFTD and 32 with SD fulfilled the criteria for the study. Of the SD patients 24 (75%) had language problems as their presenting symptom and they showed predominant left temporal atrophy whereas eight (25%) had early face recognition problems in addition to language problems and more marked right temporal atrophy. Patients’ clinical characteristics are shown in Table 1. The bvFTD and SD groups did not differ significantly with respect to gender, handedness, age at onset or duration of symptoms. Education was not consistently recorded. However, the available data indicated that the groups also did not differ in level of education (basic, qualifications at 18 years, graduate). The two groups did differ with respect to family history and genetics. A family history of dementia in a first degree relative was more common in bvFTD than SD. Likewise, an underlying genetic mutation was identified more commonly in bvFTD: it was found in 13 of 47 of bvFTD patients screened for gene mutations but none of 23 screened SD patients. Four bvFTD had mutations in the *MAPT* gene, two in the *GRN* gene and seven had repeat expansions in *C9orf72* gene. Pathological confirmation of the clinical diagnosis was available for 6/71 (8.5%) bvFTD patients and 3/32 (9.4%) SD patients.

**Table 1:** Clinical characteristics of patient groups.

	BvFTD	SD	Statistics	*p* value
Male/Female	40/31	20/12	χ^2^ = .34	*p* = .557
Handedness right/left^1^	63/7	31/0	Fisher's Exact	*p* = .097
Age at onset Mean (SD)	59.6 (9.3)	61.3 (7.0)	t = −1.01	*p* = .315
Years since symptom onset	3.0 (2.0)	3.7 (2.0)	t = −1.71	*p* = .090
Family history present	30/71 (42%)	7/32 (22%)	χ^2^ = 4.0	*p* = .046
Genetic mutation identified	13/47 (28%)	0/23 (0%)	Fisher's Exact	*p* = .003
ALS present	14/71 (20%)	0/32 (0%)	Fisher's Exact	*p* = .005
MMSE mean (SD)^2^	20.8 (7)	20.3 (6.2)	t = .30	*p* = .765
FAS^3^ mean (SD)	12.9 (11.4)	14.0 (13.3)	t = −.37	*p* = .715
Animal fluency^3^ mean (SD)	8.9 (5.5)	5.0 (4.7)	t = 3.0	*p* = .003
Weigls's blocks^4^ mean (SD)	4.8 (3.4)	7.2 (2.0)	t = −3.84	*p* < .001
Brixton errors^5^ mean (SD)	26.5 (10.2)	16.3 (6.1)	t = 4.54	*p* < .001

Missing data: ^1^1 bvFTD,1 SD patient; ^2^1bvFTD, 8 SD; ^3^3 bvFTD, 9SD; ^4^5 bvFTD, 12 SD; ^5^31bvFTD, 17 SD.

The amyotrophic lateral sclerosis (ALS) form of motor neuron disease is known to co-occur with FTD, the association being most common in bvFTD (Saxon et al., 2017). In line with those findings, ALS was present in 14 (20%) of bvFTD patients (10 with limb onset and four with bulbar onset) but none of the SD patients. BvFTD patients with ALS were older at onset (mean 64.5 years) than those without ALS (mean 58.2 years), *t* = 2.4, *p* = .02 but did not differ in other respects.

Background neuropsychological evaluation (Table 1) showed that the bvFTD and SD groups were well matched for their performance on the Mini-Mental State Examination. They also performed similarly on the FAS letter fluency task. Predictably, the bvFTD group performed significantly better than the SD group on an Animal fluency task, which makes substantial semantic demands. By contrast, they performed significantly worse on the Weigls block sorting test, an executive test of abstraction and set shifting and on the Brixton spatial anticipation test. Performance in patients with bulbar onset ALS did not differ significantly from those with limb onset ALS or bvFTD patients without ALS.

### 3.2 Naming and word comprehension performance accuracy

**Fig. 1:**
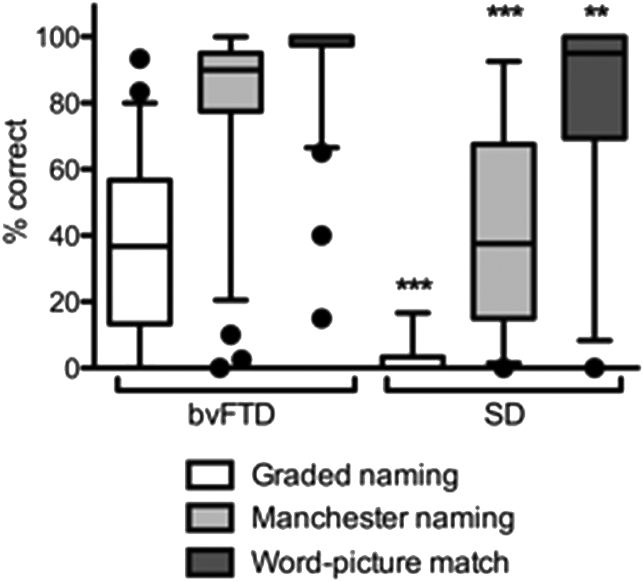
Box–Whisker plot showing naming and word-picture matching performance in bvFTD and SD, showing 5–95^th^ percentiles and outliers. Private link to Clinical Data: https://data.mendeley.com/datasets/8rv2zvsn2k/draft?a=4808ed14-5074-431e-a671-5ec3681e3fd6.

In the bvFTD group, 34/59 patients (58%) scored below the 5th percentile on the Graded Naming test, as defined by published data (Warrington, 1997), 34/71 (48%) made at least two non-viable naming errors on the undemanding Manchester naming test and 11/71 (17%) at least two errors on the word-picture matching test. This compares with 32/32 (100%) of the SD group showing impairment on both naming measures and 16/30 (53%) on word-picture matching. Two further SD patients could not undertake the word-picture matching test because of inability to understand task requirements. The distribution of scores for the two groups is shown in Fig. 1. The bvFTD group performed significantly better than the SD group on the Graded naming, Manchester naming and Manchester word-picture matching tests (Table 2).

**Table 2:** Naming and Word Comprehension Performance: accuracy and naming errors.

Test Measures		bvFTD	SD	Statistic	*p* value
Accuracy data					
Graded naming	Correct/30 mean (SD)	11.2 (7.4)	1.0 (1.8)	t = 9.9	*p* < .001
Manchester Naming	Total correct/40	33.0 (8.8)	16.0 (12.0)	t = 7.2	*p* < .001
	Biological/20	16.0 (5.1)	6.9 (6.1)	t = 7.3	*p* < .001
	Non-Biological/20	17.0 (4.1)	9.7 (6.4)	t = 5.9	*p* < .001
Word-picture match	Total correct/40	38.4 (5.3)	32.4 (11.0)	t = 2.8	*p* = .008
	Biological/20	19.2 (2.6)	15.3 (6.0)	t = 3.2	*p* = .003
	Non-Biological/20	19.2 (2.8)	18.0 (4.1)	t = 1.7	*p* = .099
Manchester Naming Test Errors (66 bvFTD and 31 SD)					
Total number of errors	Mean (SD) Range	7.0 (8.8) 0–40	24 (12) 3–40	t = −7.0	*p* < .001
Error breakdown: mean percentage of total (SD)	Semantic	25.0% (25)	23.4% (19.5)	U = 991.5, z = −.25	*p* = .80
	Superordinate	2.9% (7.5)	7.5% (11.9)	U = 698.5, z = −3.14	*p* = .002
	Associative	2.8% (10.2)	26.9% (22.8)	U = 285.5, z = −6.70	*p* < .001
	Misidentification	5.5% (13.0)	2.0% (3.1)	U = 957.0, z = −.66	*p* = .513
	Omission	12.7% (22.6)	27.0% (18.2)	U = 508.5, z = −4.25	*p* < .001
	Acceptable	51.1% (36.0)	13.3% (11.1)	U = 350.5, z = −5.28	*p* < .001
Graded Naming Errors (57 bvFTD)					
Total errors	Mean (SD)	18.4 (7.2)	–		
Percentage of total errors (SD)	Semantic	15.6% (12.6)	–		
	Superordinate	4.6% (6.3)	–		
	Associative	31.5% (20.3)	–		
	Misidentification	7.0% (7.0)	–		
	Omission	24.3% (16.8)	–		
	Acceptable	16.9% (13.1)	–		

In the Manchester naming test SD patients were disproportionately impaired relative to the bvFTD group in naming biological compared to non-biological categories (main effect of group F = 59.3, *p* < .001; main effect of task (F = 32.8, *p* < .001); group × task interaction F = 7.3, *p* = .008), and in comprehension of biological terms (main group effect F = 4.5 *p* = .04, task F = 30.7, *p* < .001 and group × task interaction F = 27.6, *p* < .001).

Comprehension performance, as measured by word-picture matching, was, unsurprisingly, superior to naming in both bvFTD and SD. Nevertheless, item-by-item comparison revealed occasional instances (19 in total across 7 bvFTD patients and 9 in total across 7 SD patients) in which items that were correctly named subsequently elicited an incorrect response on word-picture matching. One bvFTD patient, in particular, was notable. He achieved a naming score of 27/40 and comprehension score of 26/40, and there were nine instances of correct naming with impaired word-picture match selection. Most of his selection errors were visually unrelated to the target (e.g., glass for fork; skirt for trousers; blouse for boot; pumpkin for banana; donkey for elephant). This patient's performance was profoundly impaired on executive tests (he scored 0 on the simple Weigls' block sorting test).

#### 3.2.1 Correlations with demographic and background neuropsychological data

There was no correlation in either group between age at onset of symptoms at test and naming and comprehension performance (bvFTD: Graded naming r = .10, *p* = .938, Manchester naming r = −.001, *p* = .994, word picture matching r = −.028, *p* = .820; SD: Graded naming r = .189, *p* = .377, Manchester naming r = .216, *p* = .234, word picture matching r = −.036, *p* = .852. Similarly, there was no correlation with symptom duration bvFTD: Graded naming r = −.185, *p* = .164, Manchester naming r = −.192, *p* = .110, word picture matching r = .081, *p* = .506; SD: Graded naming r = −.153, *p* = .477, Manchester naming r = −.142, *p* = .440, word picture matching r = −.274, *p* = .143).

In the bvFTD group, naming and comprehension performance correlated significantly with MMSE scores (Graded naming r = .37, *p* < .004, Manchester naming r = .65, *p* < .001, word-picture matching r = .50, *p* < .001), letter fluency (Graded naming r = .42, *p* = .001, Manchester naming r = .33, *p* = .007) and category fluency (Graded naming r = .51, *p* < .001, Manchester naming r = .54, *p* < .001, word-picture matching r = .37, *p* = .002). Significant correlations were present for the SD group for the Manchester naming and word-picture matching tasks: MMSE (Manchester naming r = .58, *p* = .003, word-picture matching r = .65, *p* = .001), letter fluency (Manchester naming r = .80, *p* < .001, word-picture matching r = .65, *p* = .001) and category fluency (Manchester naming r = .76, *p* < .001, word-picture matching r = .60, *p* = .003). Graded naming scores showed weaker correlations (MMSE r = .308, *p* = 163; letter fluency r = .380, *p* = .090; category fluency r = .530,*p* = .013), likely reflecting the floor level scores present in many patients.

Naming and word comprehension performance correlated to some extent with executive test performance in the bvFTD group, significant findings arising for the Weigls blocks test (Manchester naming r_s_ = .42, *p* < .001, word-picture matching r_s_ = .40, *p* = .001) but not the Brixton test (Graded naming r = −.46, *p* = .783; Manchester naming r = −.34, *p* = .834; word-picture matching r = −.084, *p* = .600). In the SD group correlations with executive tests were non-significant (Weigls: Graded naming: Manchester naming r_s_ = .327, *p* = .159; word-picture matching r_s_ = .192, *p* = .431; Brixton errors: Graded naming r = .01, *p* = .977; Manchester naming r = .15, *p* = .606; word-picture matching r = .31, *p* = .288).

### 3.3 Naming errors

Naming errors in bvFTD encompassed the range of error types seen in SD (Table 2). Nevertheless, on the Manchester Naming test the proportional distribution of errors was not equivalent in the two groups. SD patients made a higher proportion of superordinate category substitutions, associative responses and omissions/’don't know’ responses, whereas bvFTD patients made a higher proportion of acceptable substitution errors. The proportion of semantic and misidentification errors did not distinguish the two groups. When ‘acceptable’ error responses were omitted from the analysis, SD patients continued to make a higher proportion of associative responses (U = 236, z = −5.55, *p* < .001). In bvFTD the more difficult Graded Naming test elicited a higher percentage of associative errors and omissions and reduced number of acceptable errors (Table 2), and thus was more akin to the error profile of SD.

### 3.4 Atrophy ratings

#### 3.4.1 Total cohort

SD patients had greater atrophy than bvFTD patients in left temporal pole (U = 391.0, z = −5.64, *p* < .001), right temporal pole (U = 740.0, z = −3.00, *p* = .003), left anterior hippocampus (U = 330.0, z = −6.00, *p* < .001), left entorhinal cortex (U = 497.5, z = −4.78, *p* < .001), left perirhinal cortex (U = 389.0, z = −5.64, *p* < .001), right perirhinal cortex (U = 711.5, z = −3.17, *p* = .002), left fusiform gyrus (U = 389.0, z = −5.60, *p* < .001), right fusiform gyrus (U = 743.0, z = −2.96, *p* = .003), left lateral temporal gyrus (U = 465.5, z = −5.12, *p* < .001), left superior temporal gyrus (U = 551.5, z = −4.54, *p* < .001), left mid hippocampus (U = 362.5, z = −5.82, *p* < .001), right mid hippocampus (U = 769.0, z = −2.80, *p* = .005), left posterior hippocampus (U = 448.0, z = −5.19, *p* < .001), right posterior hippocampus (U = 768.0, z = −2.79, *p* = .005) and left posterior temporal gyrus (U = 584.5, z = −4.32, *p* < .001).

SD patients also had numerically higher atrophy scores in left and right insula and other right temporal regions, which did not reach FDR-corrected statistical significance: left insula U = 859.0, z = −2.20, *p* = .028, right insula U = 1109.0, z = −22, *p* = .825; right anterior hippocampus U = 835.0, z = −2.26, *p* = .024; right entorhinal cortex U = 974.5, z = −1.21, *p* = .227; right lateral temporal gyrus U = 994.0, z = −1.07, *p* = .285; right superior temporal gyrus U = 862.5, z = −2.28, *p* = .022; right posterior temporal gyrus U = 918.5, z = −1.69, *p* = .090.

bvFTD patients had numerically higher atrophy scores in anterior brain regions but these did not reach FDR-corrected statistical significance: left anterior basal ganglia U = 1108.0, z = −.21, *p* = .831; right anterior basal ganglia U = 1108, z = −.21, *p* = .833; left orbitofrontal gyrus U = 999.0, z = −1.04, *p* = .297; right orbitofrontal gyrus U = 1061.5, z = −.56, *p* = .577; left lateral frontal gyrus U = 877.5, z = −2.11, *p* = .035; right lateral frontal gyrus U = 875.0, z = −2.09, *p* = .036; left cingulate gyrus U = 1012.0, z = −.96, *p* = .337; right cingulate gyrus U = 1078.5, z = −.442, *p* = .658.

#### 3.4.2 Matched cohort

To disentangle effects of severity per se from clinical diagnosis a sub-group of the poorest performing bvFTD patients was examined separately. This ‘severe’ group comprised 15 bvFTD patients who scored <30 on the undemanding Manchester Naming test. Their naming (mean 19.5, SD 10.6) and word picture matching (mean 33.1 SD 9.9) scores did not differ significantly from those of the SD group (naming: mean 16.0, SD 12.0; word picture match 32.4, SD 11.0): naming *t* = .97, *p* = .338; word-picture match *t* = .21, *p* = .837.

Despite being matched for severity of naming and word comprehension SD patients continued to show greater left-sided atrophy in temporal pole (U = 114.0, z = −3.2 *p* = .001), anterior hippocampus (U = 119.5, z = −2.97, *p* = .003), and lateral temporal lobe (U = 123.5, z = −2.85, *p* = .004). Differences in atrophy ratings for other regions were no longer significant: entorhinal cortex (U = 162.5, z = −1.91, *p* = .056; perirhinal cortex U = 140.5, z = −2.56, *p* = .011, fusiform gyrus (U = 170.5, z = −1.71, *p* = .087), superior temporal gyrus (U = 154.5, z = −2.14, *p* = .033) mid hippocampus (U = 140.0, z = −2.47, *p* = .0.01) and posterior hippocampus (U = 145.0, z = −2.35, *p* = .019).

### 3.5 Correlations between naming, word comprehension and atrophy ratings

Correlative analyses revealed a largely similar pattern of correlation between naming performance and atrophy ratings in bvFTD as in SD, albeit with lower correlation coefficients (Table 3). In both groups there were strong inverse correlations between naming scores and atrophy in key regions of the left temporal lobe. The principal difference between bvFTD and SD was that in SD significant correlations were more extensive within the temporal lobes, encompassing lateral temporal, insula, superior temporal and mid hippocampal regions. Notably, no significant correlations were elicited in either group with atrophy in frontal regions. A significant right-sided correlation was also elicited in SD for the Graded Naming test only in lateral temporal lobe, but this was in the opposite direction to the left-sided correlations, lower naming scores being associated with lower, rather than higher, atrophy ratings. Word comprehension, as measured by word-picture matching, elicited significant left-sided correlations in the SD group with anterior fusiform, lateral temporal, superior temporal and mid-hippocampal regions, but not anterior temporal lobe (Table 3).

**Table 3:** Association between naming and comprehension scores and atrophy.

Group	bvFTD			SD		
Task	Graded Naming	Manchester naming	Word-picture match	Graded Naming	Manchester Naming	Word-picture match
L ant BG	r_s_ = −.11 *p* = .430	r_s_ = −.19 *p* = .107	r_s_ = −.14 *p* = .249	r_s_ = .24 *p* = .254	r_s_ = −.05 *p* = .796	r_s_ = −.29 *p* = .127
R	r_s_ = −.13 *p* = .342	r_s_ = −.16 *p* = .179	r_s_ = −.14 *p* = .237	r_s_ = .37 *p* = .073	r_s_ = .11 *p* = .565	r_s_ = −.20 *p* = .281
L OFC	r_s_ = −.25 *p* = .060	r_s_ = −.09 *p* = .454	r_s_ = −.09 *p* = .459	r_s_ = −.15 *p* = .493	r_s_ = −.06 *p* = .748	r_s_ = −.18 *p* = .350
R	r_s_ = −.18 *p* = .163	r_s_ = −.04 *p* = .759	r_s_ = −.08 *p* = .495	r_s_ = .25 *p* = .249	r_s_ = .25 *p* = .175	r_s_ = −.10 *p* = .586
L LFG	r_s_ = −.15 *p* = .266	r_s_ = −.21 *p* = .086	r_s_ = −.08 *p* = .521	r_s_ = .13 *p* = .545	r_s_ = −.02 *p* = .914	r_s_ = −.10 *p* = .600
R	r_s_ = .00 *p* = .996	r_s_ = −.09 *p* = .474	r_s_ = .03 *p* = .829	r_s_ = .35 *p* = .099	r_s_ = .15 *p* = .424	r_s_ = .01 *p* = .963
L ant cing	r_s_ = −.03 *p* = .807	r_s_ = −.12 *p* = .340	r_s_ = .04 *p* = .732	r_s_ = .11 *p* = .622	r_s_ = −.02 *p* = .906	r_s_ = −.23 *p* = .218
R	r_s_ = .06 *p* = .629	r_s_ = .03 *p* = .828	r_s_ = .06 *p* = .634	r_s_ = .19 *p* = .373	r_s_ = .08 *p* = .648	r_s_ = −.14 *p* = .450
L TP	**r**_**s**_ = **-.43 *p*** = **.001**	**r**_**s**_ = **-.32 *p*** = **.006**	r_s_ = −.15 *p* = .228	**r**_**s**_ = **-.61 *p*** = **.002**	**r**_**s**_ = **-.56 *p*** = **.001**	r_s_ = −.46 *p* = .010
R	r_s_ = −.23 *p* = .076	r_s_ = −.04 *p* = .775	r_s_ = .02 *p* = .856	r_s_ = .42 *p* = .040	r_s_ = .20 *p* = .266	r_s_ = .01 *p* = .951
L ant HC R	**r**_**s**_ = **-.39 *p*** = **.002**	r_s_ = −.25 *p* = .034	r_s_ = −.17 *p* = .156	r_s_ = −.50 *p* = .013	**r**_**s**_ = **-.50 *p*** = **.004**	r_s_ = −.45 *p* = .012
	r_s_ = −.19 *p* = .161	r_s_ = −.01 *p* = .931	r_s_ = .06 *p* = .650	r_s_ = .39 *p* = .062	r_s_ = .13 *p* = .493	r_s_ = −.09 *p* = .633
L ERC	**r**_**s**_ = **-.38 *p*** = **.003**	**r**_**s**_ = **-.32 *p*** = **.007**	r_s_ = −.22 *p* = .066	r_s_ = −.48 *p* = .018	r_s_ = −.41 *p* = .019	r_s_ = −.32 *p* = .088
R	r_s_ = −.26 *p* = .047	r_s_ = −.08 *p* = .534	r_s_ = −.09 *p* = .443	r_s_ = .37 *p* = .073	r_s_ = .22 *p* = .219	r_s_ = −.04 *p* = .849
L PRC	r_s_ = −.32 *p* = .014	**r**_**s**_ = **-.34 *p*** = **.004**	r_s_ = −.19 *p* = .111	r_s_ = −.53 *p* = .008	**r**_**s**_ = **-.47 *p*** = **.007**	r_s_ = −.39 *p* = .031
R	r_s_ = −.26 *p* = .044	r_s_ = −.19 *p* = .121	r_s_ = −.13 *p* = .272	r_s_ = .49 *p* = .015	r_s_ = .26 *p* = .158	r_s_ = −.01 *p* = .978
L ant fus	**r**_**s**_ = **-.36 *p*** = **.006**	**r**_**s**_ = **-.34 *p*** = **.004**	r_s_ = −.18 *p* = .132	**r**_**s**_ = **-.68 *p*** < **.001**	**r**_**s**_ = **-.65 *p*** < **.001**	**r**_**s**_ = **-.62 *p*** < **.001**
R	r_s_ = −.22 *p* = .088	r_s_ = −.15 *p* = .219	r_s_ = −.11 *p* = .374	r_s_ = .51 *p* = .010	r_s_ = .25 *p* = .172	r_s_ = .02 *p* = .899
L lat temp	r_s_ = −.08 *p* = .557	r_s_ = −.12 *p* = .337	r_s_ = −.12 *p* = .329	r_s_ = −.42 *p* = .039	**r**_**s**_ = **-.56 *p*** = **.001**	**r**_**s**_ = **-.53 *p*** = **.003**
R	r_s_ = −.03 *p* = .835	r_s_ = −.07 *p* = .587	r_s_ = −.04 *p* = .759	**r**_**s**_ = **.62 *p*** = **.001**	r_s_ = .32 *p* = .079	r_s_ = .05 *p* = .797
L insula	r_s_ = −.22 *p* = .101	r_s_ = −.15 *p* = .213	r_s_ = −.19 *p* = .126	r_s_ = −.48 *p* = .018	**r**_**s**_ = **-.48 *p*** = **.006**	r_s_ = −.44 *p* = .015
R	r_s_ = −.04 *p* = .744	r_s_ = .06 *p* = .645	r_s_ = .04 *p* = .722	r_s_ = .54 *p* = .007	r_s_ = .29 *p* = .107	r_s_ = .02 *p* = .925
L sup temp	r_s_ = −.05 *p* = .695	r_s_ = −.14 *p* = .233	r_s_ = −.15 *p* = .229	r_s_ = −.47 *p* = .021	**r**_**s**_ = **-.52 *p*** = **.002**	**r**_**s**_ = **-.49 *p*** = **.006**
R	r_s_ = −.07 *p* = .582	r_s_ = .03 *p* = .785	r_s_ = −.16 *p* = .198	r_s_ = .49 *p* = .014	r_s_ = .12 *p* = .523	r_s_ = −.11 *p* = .551
L mid HC	r_s_ = −.20 *p* = .135	r_s_ = −.22 *p* = .067	r_s_ = −.23 *p* = .053	r_s_ = −.38 *p* = .070	**r**_**s**_ = **-.46 *p*** = **.009**	**r**_**s**_ = **-.51 *p*** = **.004**
R	r_s_ = −.18 *p* = .177	r_s_ = −.09 *p* = .468	r_s_ = −.17 *p* = .168	r_s_ = .35 *p* = .090	r_s_ = .15 *p* = .430	r_s_ = −.16 *p* = .414
L pos HC	r_s_ = −.12 *p* = .354	r_s_ = −.12 *p* = .312	r_s_ = −.16 *p* = .179	r_s_ = −.36 *p* = .086	r_s_ = −.41 *p* = .020	r_s_ = −.42 *p* = .020
R	r_s_ = −.02 *p* = .884	r_s_ = .05 *p* = .657	r_s_ = −.11 *p* = .363	r_s_ = .42 *p* = .043	r_s_ = .20 *p* = .263	r_s_ = −.05 *p* = .809
L pos temp	r_s_ = −.15 *p* = .267	r_s_ = −.17 *p* = .153	r_s_ = −.19 *p* = .114	r_s_ = −.25 *p* = .242	r_s_ = −.33 *p* = .067	r_s_ = −.27 *p* = .144
R	r_s_ = −.13 *p* = .321	r_s_ = −.06 *p* = .629	r_s_ = −.18 *p* = .133	r_s_ = .52 *p* = .010	r_s_ = .17 *p* = .358	r_s_ = −.01 *p* = .959

L = left, R = right. ant = anterior, mid = middle, pos = posterior, lat = lateral, sup = superior, BG = basal ganglia, OFC = orbitofrontal cortex, LFG = lateral frontal gyrus, cing = cingulate, TP = temporal pole, HC = hippocampus, ERC = entorhinal cortex, PRC = perirhinal cortex, fus = fusiform, temp = temporal.

Significant correlations corrected for multiple comparisons are shown in bold.

Interestingly, separate analysis for biological and non-biological items in the Manchester naming task in the SD group showed that the positive correlations with left lateral temporal, anterior and mid hippocampal and superior temporal atrophy (Table 3) were largely driven by naming performance for biological items. For non-biological items only the correlations with temporal pole (r_s_ = −.51, *p* = .003) and fusiform atrophy (r_s_ = −.51, *p* = .003) remained statistically significant. The pattern of correlations for word comprehension of biological and non-biological terms, measured by the Manchester word-picture matching task, was more uniform. Correlations, in line with those shown in Table 3, were present for both biological and non-biological terms, the only exception being that a correlation with superior temporal lobe atrophy reached significance for biological (r_s_ = −.53, *p* = .005) but not non-biological terms (r_s_ = −.47, *p* = .013).

### 3.6 Correlations between naming errors and atrophy ratings

#### 3.6.1 bvFTD

In bvFTD, no significant correlations were elicited between *semantic* errors in naming and atrophy ratings. There were strong positive correlations between the number of *superordinate* category errors on the Graded naming test and atrophy ratings in both left and right hemispheres: left temporal pole r_s_ = .38, *p* = .004, left anterior hippocampus r_s_ = .40, *p* = .002, right anterior hippocampus r_s_ = .40, *p* = .002, left entorhinal cortex r_s_ = .44, *p* = .001, right entorhinal cortex r_s_ = .38, *p* = .004, left perirhinal cortex r_s_ = .36, *p* = .004, right perirhinal cortex r_s_ = .41, *p* = .002, left fusiform gyrus r_s_ = .47, *p* < .001, right fusiform gyrus .39, *p* = .003, left lateral temporal lobe r_s_ = .36, *p* = .006, left insula r_s_ = .38, *p* = .004, left superior temporal gyrus r_s_ = .48, *p* < .001, right superior temporal gyrus r_s_ = .40, *p* = .002, left posterior temporal gyrus r_s_ = .37, *p* = .005. A similar although less extensive pattern obtained for the easy Manchester naming test: superordinate category errors correlated positively with atrophy of the left temporal pole r_s_ = .38, *p* = .001, left perirhinal cortex r_s_ = .34, *p* = .003 and left fusiform gyrus r_s_ = .40, *p* = .001.

*Associative* errors correlated positively with atrophy of the left temporal pole on Graded naming (r_s_ = .43, *p* = .001) and to a lesser extent on Manchester naming (r_s_ = .33, *p* = .005).

O*missions* on the Graded Naming test, constituting ‘don't know’ and generic responses insufficient to convey identifying information did not correlate with atrophy ratings. However, omission errors on the Manchester naming test correlated with left-sided atrophy in entorhinal cortex, r_s_ = .34, *p* = .004, perirhinal cortex r_s_ = .32, *p* = .007, fusiform r_s_ = .39, *p* = .001 and mid hippocampus r_s_ = .35, *p* = .003.

*Misidentification* errors in bvFTD were rare and elicited no significant correlations. Similarly, no significant correlations were elicited for *Acceptable* errors, which accords with prediction given that such errors are assumed to be non-pathological.

#### 3.6.2 SD

In the SD group, most patients performed at floor level on the Graded Naming test, so it was necessary in some instances to discontinue the test before completion. Error analysis was therefore carried out for the undemanding Manchester naming test only. *Semantic* errors showed positive correlations with atrophy in the right posterior hippocampus, r_s_ = .54, *p* = .002. No correlations were elicited between *superordinate* category errors and atrophy.

*Associative* errors correlated positively with atrophy of the left temporal pole, r_s_ = .52, *p* = .003. There were also inverse correlations with right-sided atrophy in lateral temporal lobe, r_s_ = −.56, *p* = .001, insula r_s_ = −.50, *p* = .004, mid hippocampus, r_s_ = −.51, *p* = .003 and posterior hippocampus r_s_ = −.55, *p* = .001.

*Omissions* were positively correlated with left-sided atrophy of fusiform gyrus r_s_ = .55, *p* = .001. There were trends towards a correlation with anterior hippocampal (r_s_ = .46, *p* = .009), lateral temporal (r_s_ = .49, *p* = .005) and superior temporal (r_s_ = .46, *p* = .008) lobe atrophy, but these did not reach FDR corrected levels of significance.

Like bvFTD, *misidentification* errors in SD and *acceptable* substitution errors showed no significant correlations with atrophy.

### 3.7 Within-group variation

Within the bvFTD group, participants were divided into those performing above and below the mean naming score. Imaging ratings for the two sub-groups differed for left-sided atrophy of temporal pole (U = 294.0 z = −2.80, *p* = .005), entorhinal cortex (U = 258.0, z = −3.26, *p* = .001), perirhinal cortex (U = 274.0, z = −3.02, *p* = .003), fusiform gyrus (U = 247.0, z = −3.43, *p* = .001) and mid hippocampus (U = 290.5, z = −2.87, *p* = .004). Atrophy in frontal regions and right hemisphere elicited no significant differences.

There were no performance differences on naming and word comprehension tasks between bvFTD patients with and without accompanying ALS. Similarly, there were no differences in ratings of atrophy. BvFTD with a positive family history of dementia did not differ from those without a family history in their test performance or image ratings and bvFTD patients with an identified genetic mutation did not differ in their test performance or imaging ratings from those with no mutation. When patients with identified mutations only were considered there were hints at differences. Patients with *MAPT* mutations achieved numerically lower naming scores than those with other mutations but in view of the small group size differences were not statistically significant. Patients with *MAPT* mutations showed a trend towards more severe atrophy in left entorhinal cortex (U = 2.0, z = −2.68, *p* = .007) and fusiform gyrus (U = 1.5, z = −2.72, *p* = .006) and right perirhinal cortex (U = 1.5, z = −2.67,*p* = .007) than patients with mutations in the *GRN* and *C9orf72* gene and in right fusiform gyrus compared to bvFTD patients with no gene mutation (U = 13.0, z = −2.80, *p* = .005).

## 4 Discussion

In this large cohort of bvFTD patients naming problems were a prominent feature of many, although not all, patients (around 50%). A smaller proportion (around 17%) exhibited impairment on an undemanding word-picture matching test, indicating that deficits were not confined to naming but also involved comprehension of words. These findings point to a core semantic deficit, at least in a proportion of bvFTD patients, supporting others’ claims (Hardy et al., 2015).

Predictably, direct comparison between bvFTD and a cohort of SD patients revealed less severe and ubiquitous naming and word comprehension deficits in bvFTD. It also revealed less severe atrophy in temporal lobe structures in bvFTD, in keeping with previous reports (Kipps et al., 2007). Nevertheless, the pattern of correlations between naming and atrophy ratings in the two clinical groups showed similarities, with strong correlations being elicited in both groups for atrophy ratings in the left anterior temporal lobe and fusiform gyrus. The principal group difference was the more extensive number of regions within the left temporal lobe yielding correlations in the SD group and the presence of inverse correlations in SD in the right hemisphere. SD, but not bvFTD, patients showed disproportionate impairment in naming and comprehension of biological items (animals, fruit and vegetables), and it was those items that largely drove the correlations between naming and atrophy in structures beyond the temporal pole and fusiform gyrus.

A pertinent question at the outset was the degree to which executive impairments and associated frontal lobe atrophy might influence naming performance in bvFTD. The significant correlation between naming and Weigls block sorting scores in bvFTD but not SD suggests that an executive contribution might have a role. Moreover, examination of individual profiles suggests a ‘frontal’ contribution in at least some patients. It would be expected that correctly named items should also be selected correctly on word-picture matching, notwithstanding occasional visually-based errors. Yet in one bvFTD patient in particular there were a relatively large number of instances of correct naming with impaired comprehension performance. It is likely that executive failures accounted for the incorrect response selections in that patient. A feature of bvFTD is lack of adherence to task rules. In a forced-choice condition patients may sometimes base responses on personal preference or idiosyncratic criteria or else respond randomly due to inattention. It is noteworthy that the bvFTD patient showed severely impaired performance on the Weigls test, achieving a score of 0.

Nevertheless, in the bvFTD group as a whole, there was no correlation with one nonverbal test of executive function (Brixton spatial anticipation test). Moreover, unlike some earlier studies (Grossman et al., 2004, Hardy et al., 2015), no significant correlations were present in bvFTD between naming scores and atrophy ratings in frontal lobe regions. It seems likely that potential ‘frontal’ executive contributions to naming, likely present in at least some patients, are overshadowed, in this study, by strong primary naming impairments in a proportion of patients, linked to atrophy in temporal regions. This has implications for our understanding of FTD. FTD patients are typically classified according to prototypical clinical phenotypes: bvFTD, SD and non-fluent progressive aphasia. Yet, patients may not all fall neatly into those discrete syndromic categories. The present findings reinforce the view (Hardy et al., 2015, Harris et al., 2016) that there is clinical overlap between clinical syndromes of FTD.

What are the factors that govern the presence of naming and comprehension impairment in bvFTD? Strong correlations between naming and MMSE scores suggest that stage of illness is an important contributor to individual differences in performance. Yet, this relationship is not straightforward. The MMSE makes language demands and so is not an entirely independent marker of disease severity. There was no correlation between naming performance and duration of symptoms. There are likely to be other contributory factors to within-group differences. Studies of ALS (Goldstein and Abrahams, 2013, Strong et al., 2017, Taylor et al., 2013) and FTD-ALS (Saxon et al., 2017) have highlighted the importance of language problems as a key part of patients’ cognitive disorder, raising the potential for differences in bvFTD patients with and without ALS. Nevertheless, in this study, the co-occurrence of ALS did not correlate with the magnitude of naming disorder. In genetic bvFTD it was expected, in line with previous evidence (Hardy et al., 2015, Rohrer et al., 2015, Snowden et al., 2015), that patients with a *MAPT* mutation would show more severe naming problems than those with other mutations. Numerical test scores were indeed in the predicted direction although the small numbers within each genetic group meant that statistical evidence was lacking. What is clear is that naming and word comprehension performance reflects the magnitude of atrophy in key temporal lobe structures and that this varies across the patient cohort.

An intriguing theoretical challenge in recent decades has come from the profound loss of knowledge in SD occurring in the context of severe anterior temporal lobe atrophy. The anterior temporal lobe's seemingly pivotal importance in underpinning semantic memory led to its designation as a ‘semantic hub’ (Patterson et al., 2007). Yet, this anterior temporal emphasis is contrary to traditional assumptions, supported by lesion and neuroimaging studies (Bonilha et al., 2017, Chao et al., 1999, Hart and Gordon, 1990, Martin and Chao, 2001, Thompson-Schill, 2003) of an association between loss of word comprehension and damage to more posterior temporal lobe regions. The current study addresses such apparent contradictions. Our findings in SD are in accord with arguments that damage to left anterior temporal lobe gives rise to naming impairment but not loss of conceptual knowledge (Bi et al., 2011, Busigny et al., 2015, Mesulam et al., 2013). Whereas naming in SD correlated strongly with atrophy of the temporal pole, word comprehension, measured by word-picture matching, showed stronger correlations with more posterior temporal structures.

In most bvFTD patients performance on word-picture matching approached ceiling levels, so that correlative data are uninformative. Nevertheless, the naming data in bvFTD are instructive. bvFTD patients who performed above or below average on the Manchester naming task were distinguished by the severity of atrophy in left-sided temporal structures, which included anterior temporal lobe, pointing to the importance of these structures for naming. However, when bvFTD and SD groups were matched for naming and word comprehension performance, by excluding better performing bvFTD patients, anterior temporal lobe/temporal pole atrophy remained significantly greater in the SD group. By contrast, differences in atrophy for entorhinal and perirhinal cortex, fusiform gyrus, superior temporal gyrus and mid and posterior hippocampus, which had been present for SD comparisons with the full bvFTD cohort, were no longer significant. Such findings argue for the critical importance of these latter structures for semantic memory. The anterior temporal lobe/temporal pole may not be key.

In the present study, the strongest correlations with naming and word comprehension scores in the SD group were atrophy ratings in fusiform gyrus. This is a relevant finding because it complements other data. Mion et al. (2010) argued that the left anterior fusiform gyrus atrophy is a better predictor of naming performance in SD than anterior temporal lobe atrophy. Davies et al. (2009) showed, in a voxel-based morphometric study, that fusiform atrophy best distinguished SD from controls. Libon et al. (2013) related performance of SD patients on a category membership task to atrophy in left fusiform gyrus. A recent study of patients with epilepsy using electrocorticography, functional MRI and cortical stimulation (Forseth et al., 2018) revealed that only in the middle fusiform gyrus did direct cortical stimulation disrupt naming tasks whilst still preserving the ability to repeat sentences. The authors proposed a model in which a distinct neuroanatomical substrate in middle fusiform gyrus provides access to object semantic information. A key point about all these data is that they move away from the notion of the temporal polar region as having a privileged role in semantic memory.

The analysis of errors complements the accuracy data. In keeping with previous findings (Snowden et al., 2018), in the SD group associative-type errors correlated with temporal polar atrophy whereas omission errors correlated with atrophy in more posterior temporal lobe structures. The production of associative errors, such as “in Australia” in response to a picture of a kangaroo, implies that the patient has some conceptual understanding of the object that they cannot name. By contrast, omission errors, such as the response “I don't know what that is” suggest a fundamental loss of conceptual knowledge. A similar pattern of correlations was seen in bvFTD. Whereas superordinate category substitutions and associative-type errors correlated with anterior temporal lobe atrophy omission errors did not, significant correlations being elicited for more posterior temporal structures. The error analysis revealed other notable characteristics in SD. Semantic errors, such as “dog” for tiger, which are the hallmark of SD, were associated with atrophy in posterior regions of the right temporal lobe. By contrast, associative errors showed inverse correlations with right temporal atrophy. Such disparities are likely to result from asymmetries of atrophy within the SD group: left predominant in 24 patients and right-sided predominance in 8 and, as argued previously (Snowden et al., 2018), may reflect the differential contribution of the two hemispheres to visual perceptual and associative aspects of semantic knowledge. The findings attest to the importance of error analysis in the evaluation of patients' naming disorder, as argued by others (Rohrer et al., 2008). They challenge the notion of the temporal polar region as a semantic hub.

What then of the status of the hub? Should it be located elsewhere, such as the fusiform gyrus, as argued by some (Forseth et al., 2018, Mion et al., 2010), or should the notion of a hub be revised or discarded? The idea of a “hub” is central to the hub-and- spoke model of semantic memory (Chen et al., 2017, Lambon Ralph et al., 2017, Patterson et al., 2007). The model, which acknowledges the generally accepted view that semantic memory involves a widely distributed network, includes both modality specific components, involving brain regions outside the anterior temporal lobes (the spokes) and a central component, the anterior temporal lobes (the hub), in which representations abstract away from modality-specific attributes to enable generalisations to be made across concepts that may have similar significance but differing sensory properties. The need for a central hub is not universally accepted. Some authors have argued for a distributed multimodal semantic system (or systems) that does not involve a hub (Barsalou, 2008, Kiefer and Pulvermüller, 2012, Martin, 2007, Martin, 2016, Meteyard et al., 2012, Pulvermüller, 2013). The brain regions involved correspond (or at least overlap) with those involved in sensory perception, action and language and object concepts emerge from weighted activity within property-based brain regions. Other authors have proposed that generalisations across concepts might arise through the structuring influence of language rather than via a domain-general hub (Gainotti, 2017).

The strength of correlations between naming and word comprehension and left fusiform atrophy would be compatible with the argument (Forseth et al., 2018) that this region is important in providing access to object semantic information. But should it be considered a hub? There were strong correlations too with other brain regions. Moreover, the different pattern of correlation in SD for the naming of biological and non-biological items suggests differential involvement of different brain structures rather than a single core structure. Furthermore, damage to a semantic hub, which implies a difficulty in generalising across concepts, might be expected to lead to semantic errors, yet there was no significant correlation between the presence of semantic errors and fusiform atrophy.

In a recent formulation, Woollams and Patterson (2018) have reframed the hub and spokes respectively in terms of “representations” and “connections” and placed emphasis on the importance of connections in semantic cognition. We too have emphasised the importance of connectivity (Snowden et al., 2018). We drew attention to the fact that in SD the characteristic pathological changes are elongated dystrophic neurites, which traverse the entire depth of the cerebral cortical ribbon (Mann & Snowden, 2017). That is, the predominant neurobiological problem is in the connections between neurones rather than within the cell body itself. We argued (Snowden et al., 2018) that whilst the anterior temporal lobes are indisputably the primary site of pathology in SD it is only when the pathology has evolved sufficiently to result in widespread secondary disruption of function of ventral pathways that the profound semantic loss characteristic of SD becomes apparent. The implication is that there may be no domain-general hub in which concepts are *represented* and which can be disrupted by selective damage. The semantic loss may be a product of widespread loss of *connections* across the semantic network. The current findings in bvFTD would be compatible with that notion. Future work needs to examine directly the counter-argument that semantic loss becomes evident only and specifically when the atrophy extends to fusiform gyrus.

A strength of the present study is the large cohort of bvFTD patients and the opportunity to make a direct comparison with naming in SD. A limitation of the study is the undemanding nature of the word-picture matching test. This comprehension test was chosen because its widespread use optimised sample size and because it offered the potential for direct comparison between naming and word comprehension. Nevertheless, the test is insensitive to subtle levels of comprehension impairment. Therefore, whereas the study provides robust evidence of word comprehension impairment in some bvFTD patients it does not have the scope to determine the frequency with which bvFTD patients are affected. A further potential limitation is that imaging was largely based on clinical scans, which were carried out in different hospitals using different data acquisition protocols so that voxel-based morphometric analysis of images was not viable. Nevertheless, the visual rating scale used is appropriate for the clinical population and has proven validity (Harper et al., 2016). The multiple statistical comparisons necessitate caution in their interpretation. However, false discovery rate controls were applied. Moreover, the data are robust, internally consistent and consonant with independent findings using voxel-based morphometry, suggesting that significant findings are unlikely to be spurious. Only a small proportion of patients had post-mortem confirmation of diagnosis, so that the possibility of alternative diagnoses cannot be unequivocally ruled out. Nevertheless, the presence of genetic mutations in 28% of the bvFTD cohort increases the likelihood of a frontotemporal lobar degeneration pathology. Moreover, an earlier study of clinico–pathological correlations (Snowden et al., 2011) elicited a high degree of clinical diagnostic accuracy.

In conclusion, the naming and word comprehension data in bvFTD inform both clinical understanding of the condition and theoretical understanding of the neural underpinnings of semantic cognition. Problems in naming are a prominent feature of bvFTD in many patients. The naming disorder is additional to and not solely a secondary consequence of patients’ executive disorder. Moreover, commonalities with findings in SD highlight the clinical overlap between bvFTD and SD. The correlative data reinforce the notion of a widespread semantic network underpinning naming and word comprehension and challenge the status of the anterior temporal lobes as a semantic hub.

## Data repository and data access

Clinical data are available at https://doi.org/10.17632/8rv2zvsn2k.

Published tests materials are copyrighted and are available from the publishers (Graded naming test: Cambridge Cognition (http://www.cambridge.cognition.com/); Brixton test: Pearson Assessment (http://www.pearsonclinical.co.uk/).

Brain scans are NHS clinical scans, which contain identifiable personal information. They cannot be made publically available for ethical reasons to protect patient confidentiality. Access would require special permission from the relevant hospital Trusts. Queries regarding access should be directed to Dr. Christopher Kobylecki (christopher.kobylecki@manchester.ac.uk).

## CRediT authorship contribution statement

**Julie S. Snowden:** Conceptualization, Data curation, Formal analysis, Methodology, Project administration, Writing - original draft. **Jennifer M. Harris:** Data curation, Writing - review & editing. **Jennifer A. Saxon:** Investigation, Writing - review & editing. **Jennifer C. Thompson:** Methodology, Writing - review & editing. **Anna M. Richardson:** Writing - review & editing. **Matthew Jones:** Methodology, Writing - review & editing. **Christopher Kobylecki:** Data curation, Methodology, Project administration, Writing - review & editing.
