# Supplementary material for: Naming and conceptual understanding in frontotemporal dementia
Source: Cortex. 2019 Nov;120:22–35. doi: 10.1016/j.cortex.2019.04.027 (PMC6838679; doi:10.1016/j.cortex.2019.04.027)
Supplement: Multimedia component 1 [file mmc1.docx]

**Comparison of the Kipps and Davies Rating Scales**

On the Kipps scale SD patients showed greater atrophy than bvFTD patients in the left anterior temporal (U=391.0, z=-5.64, p<.001), right anterior temporal (U=756.0, z=-2.88, p=.004, left posterior temporal (U=335.0, z=-6.02, p<.001), and right posterior temporal (U=734.5, z=-3.06, p=.002) lobes. There was a trend for greater atrophy in bvFTD in the left frontal lobe (U=800.5, z=-2.20, p=.009), but this did not reach FDR-controlled levels of significance.

Examination of the relationship between the two rating scales elicited a perfect or near perfect mapping between atrophy ratings in the temporal pole on the Davies scale and anterior temporal lobe on the Kipps scale (left: r_s_= 1.0, p<0.001; right: 0.995, p<0.001) and between mid-hippocampal atrophy on the Davies scale and posterior temporal atrophy on the Kipps scale (left: r_s_=0.99, p<0.001; right: r_s_=0.99, p<0.001). There were additional strong associations (correlation coefficients above 0.80) between posterior temporal atrophy on the Kipps scale and posterior hippocampal atrophy on the Davies scale (left: r_s_=0.89, p<0.001; right: r_s_=0.89, p<0.001).

References

Davies, R.R., Scahill, V.L., Graham, A., Williams, G.B., Graham, K.S. & Hodges, J.R. (2009). Development of an MRI rating scale for multiple brain regions: comparison with volumetrics and with voxel-based morphometry. *Neuroradiology*, 51, 491-503. https://doi.org/10.1007/s00234-009-0521-z.

Kipps, C.M., Davies, R.R., Mitchell, J., Kril, J.J., Halliday, G.M., & Hodges, J.R. (2007). Clinical significance of lobar atrophy in frontotemporal dementia: application of an MRI visual rating scale. *Dementia and Geriatric Cognitive Disorders*, 23(5), 334-342. https://doi.org/10.1159/000100973.

Snowden JS, Harris JM, Thompson JC, Kobylecki C, Jones M, Richardson AM, & Neary D. (2018). Semantic dementia and the left and right temporal lobes. *Cortex* 107, 188-203. https://doi.org/10.1016.j.cortex.2017.08.024.
